# Bone–cartilage crosstalk: a conversation for understanding osteoarthritis

**DOI:** 10.1038/boneres.2016.28

**Published:** 2016-09-20

**Authors:** David M Findlay, Julia S Kuliwaba

**Affiliations:** 1 Discipline of Orthopaedics and Trauma, The University of Adelaide, Adelaide, South Australia 5005, Australia

## Abstract

Although cartilage degradation is the characteristic feature of osteoarthritis (OA), it is now recognized that the whole joint is involved in the progression of OA. In particular, the interaction (crosstalk) between cartilage and subchondral bone is thought to be a central feature of this process. The interface between articular cartilage and bone of articulating long bones is a unique zone, which comprises articular cartilage, below which is the calcified cartilage sitting on and intercalated into the subchondral bone plate. Below the subchondral plate is the trabecular bone at the end of the respective long bones. In OA, there are well-described progressive destructive changes in the articular cartilage, which parallel characteristic changes in the underlying bone. This review examines the evidence that biochemical and biomechanical signaling between these tissue compartments is important in OA disease progression and asks whether such signaling might provide possibilities for therapeutic intervention to halt or slow disease development.

## Introduction

Osteoarthritis (OA) manifests as degradation and loss of the articular cartilage, but typically involves all tissues of the joint. Although this review focusses on crosstalk between the subchondral bone and articular cartilage in the initiation and progression of OA, likely inputs from each of the other tissues involved in the joint structure is acknowledged.

Consideration of the crosstalk between bone and cartilage as a factor in OA initiation and progression raises several key questions. First, is it feasible that these tissue compartments communicate? If so, is this communication important in health and disease? Is the communication biomechanical or biochemical, or both? Second, what are the changes that take place in bone and cartilage during the development of OA and how might these changes in either tissue affect the other? Third, if there is a crosstalk between bone and cartilage that is important in the development of OA, could treatments for OA be directed to modifying this crosstalk by inhibiting the disease-related changes in these tissues? Should therapies be targeted to bone or cartilage?

The evidence that will be reviewed below comes largely from *in vitro* and animal experiments. For the most part, the relevance of these data for human OA is not known, and translation of treatments that has been effective in animals has been disappointing in humans so far. Our understanding of human OA is enriched by population studies, non-invasive imaging, and examination of joint tissues at end-stage disease. It is hoped that higher-resolution imaging studies, with a better appreciation of what these mean at the tissue level, will drive this field forward in terms of developing new treatment strategies, and better identification of patients in whom these treatments might be effectively applied.

## Feasibility of bone–cartilage crosstalk

Although the articular cartilage overlies, and is in intimate contact with the underlying subchondral bone, molecular crosstalk between osteoblasts/osteocytes and chondrocytes *in vivo*, particularly in human joints, is unproven. The traditional view has been that the calcified layer of the cartilage, immediately above the subchondral plate, and the subchondral plate act as impenetrable barriers. However, there is a body of evidence suggesting that these tissues can communicate. For example, the interface between the subchondral bone and calcified cartilage contains numerous vascular canals.^1^ Duncan *et al.*^2^ described holes in the subchondral plate, which were largely located beneath the area that is usually covered by the meniscus, a consistent finding for healthy medial tibial plateaus of knee joints. Some of these holes appeared to penetrate the subchondral plate and connect with the marrow space. These findings are consistent with more recent descriptions of the human chondro-osseous junction as being more complex than previously appreciated, with uncalcified cartilage frequently dipping through the calcified cartilage into bone and marrow spaces,^3^ again suggesting a potential route for molecular diffusion between the two compartments. Imhof *et al.*^4^ described the dense subchondral vasculature in close proximity to the cartilage and the micro-channels that penetrate the subchondral bone and permit communication between the bone and cartilage. These authors claimed that >50% of the glucose, oxygen, and water requirements of cartilage are provided by perfusion from these subchondral vessels. Consistent with this, experimentally induced hypoxia of the femoral head led to cell death in both the bony epiphysis and in the deep layer of the overlying cartilage.^5^ Work by Pan *et al.*,^6^ using fluorescent dyes, showed that small molecules (for example, sodium fluorescein, 376 Da) could diffuse readily between the bone marrow and the articular space in mouse joints. These observations show, at least in small mammals, the possibility of direct signaling between the subchondral bone and articular cartilage, at least for small molecules, with the suggestion that cartilage and bone form a functional unit, both mechanically and biochemically, which may have a role in joint homeostasis and disease. More relevant perhaps for larger mammals, including humans, are experiments performed in healthy horse metacarpal joints. Experiments in joints obtained immediately after death demonstrated that, in contrast to previous reports, the tidemark and mineralized cartilage are permeable to low-molecular-weight solutes.^7^ By perfusing the joint *ex vivo* from the subchondral side with fluorescent dyes (~400 Da), it was observed that tracer penetrated through the cartilage and after 1.5 h was present in the synovial fluid, suggesting that dye entry was from the subchondral micro-circulation. These experiments did not address the possibility of exchange of larger molecules such as cytokines and to explore this further the contribution of cyclic loading of the joint needs to be considered. To do so, O’Hara *et al.*^8^ performed cyclic loading of human femoral head articular cartilage and found that it increased diffusion through the cartilage of large molecules, such as human serum albumin (66.5 kDa), but did not influence the diffusion of small solutes. More recently, diffusion of insulin-like growth factor (7.6 kDa) through the cartilage *ex vivo* was shown to be significantly enhanced by cyclic loading.^9,10^ Similarly, larger molecules (12.3 kDa) than previously thought able to traverse osteocyte canaliculi have been shown to do so and this transport is increased by bone loading.^11^ Thus, there are likely to be both vascular and other means, including via the osteocyte lacuna–canalicular network of bone, for signaling molecules to traverse between the bone and cartilage.

In OA, there is increased ability for fluid movement between bone and cartilage, and therefore, presumably, for trafficking of humoral mediators. Hwang *et al.*^12^ found that the hydraulic conductance (that is, the ease of fluid flow) of human osteochondral plugs increased in association with increasing cartilage erosion and subchondral bone plate thickness and vascularity. The authors noted that alteration of fluid flow across the cartilage–bone interface could affect the mechanical and chemical environment in ways that promote the progression of OA. Several studies have suggested that there is increased porosity of the subchondral plate in OA, which may enhance the interaction between the bone and cartilage compartments. A large increase in subchondral plate porosity was shown during disease development in a mouse model of instability-induced knee OA,^13^ and Iijima *et al.*^14^ also found that subchondral plate porosity increased during disease progression in a rat model of post-traumatic knee OA. The increase in plate porosity and consequent eruption of blood vessels through the plate and into the calcified cartilage seems to be due to the increased osteoclastic activity.^12,13^ The molecular mechanisms involved in the increased angiogenesis in OA and the reduced ability of the cartilage to resist vascular invasion have been well reviewed elsewhere.^15^ In the Iijima study cited above,^14^ the increased porosity co-localized with the point of mechanical load during ambulation. We also found that bone marrow lesions in the subchondral bone of human tibiae most frequently co-located with predicted zones of maximal loading,^16^ and that bone marrow lesions associate in turn with increased porosity of the subchondral plate,^16^ and with microcrack accumulation in the plate and calcified cartilage (Kuliwaba *et al.*, unpublished). Bone marrow lesions are discussed in more detail below, but it is relevant here to cite reports that describe perfusion abnormalities in the subchondral bone in both animal models of OA^17,18^ and human OA, in particular in zones of the subchondral bone identified as bone marrow lesions.^19^ If crosstalk between the bone and cartilage is dependent on the diffusion of factors from the subchondral vasculature, reduced perfusion will have implications for this crosstalk.

## Evidence for osteoblast–chondrocyte crosstalk

### At the cell and tissue level

There is abundant evidence that cells in the bone, particularly osteoblasts and osteocytes, and those in the cartilage, chondrocytes, alter their behavior in OA. This altered behavior, in particular for bone cells, is described in more detail below. There are a range of environmental cues that these changes in behavior could be in response to, which include biomechanical and biochemical. It is thought that overloading is an important driver of OA, whether due to obesity or altered joint biomechanics, and both chondrocytes^20^ and osteoblast/osteocytes^21,22^ have well-developed mechano-sensing abilities. Other cues, perhaps at different stages of the disease, include damage to the matrix of the bone and cartilage, in which these cells reside,^23^ perhaps also as a consequence of inappropriate loading of the joint; hypoxia, perhaps due to vascular pathology in the joint;^24^ and inflammation, which when prolonged drives a catabolic agenda in the bone and cartilage.^25^ It is also possible that cells in the bone and cartilage compartments exert influence on each other and there is *in vitro* and *ex vivo* evidence that they can do this (described below). However, as discussed above, although small molecules are able to traverse by diffusion between the bone and cartilage, it is not clear whether this applies to larger signaling molecules.

There is accumulating *ex vivo* and *in vitro* evidence that events in the subchondral bone can affect the behavior of the overlying cartilage. In an experiment with cultured bovine osteochondral explants, Amin *et al.*^26^ reported that articular cartilage in the absence of subchondral bone showed increased chondrocyte death after 7 days, mainly in the superficial zone of the cartilage. In contrast, when the subchondral bone was included in the culture, either excised from the cartilage or remaining attached, the chondrocytes remained largely viable. It was speculated that soluble chondrocyte survival factor(s) were released from the subchondral bone. Sanchez *et al.*^27,28^ have described a co-culture system, in which osteoblasts derived, respectively, from ‘sclerotic’ (more severely osteoarthritic) or ‘non-sclerotic’ regions of the subchondral bone in human patients with knee OA were separated by a membrane from chondrocytes that were derived from the articular knee cartilage and grown in alginate beads. Compared with chondrocytes cultured alone, chondrocytes in the presence of ‘sclerotic’ osteoblasts, but not ‘non-sclerotic’ osteoblasts, showed reduced production of the cartilage matrix protein aggrecan and increased expression of the cartilage degrading enzyme, matrix metalloproteinase (MMP)3 and MMP13. In more recent experiments, these authors confirmed reduced expression of aggrecan messenger RNA (mRNA) by chondrocytes in co-culture with ‘sclerotic’ osteoblasts.^28^ In addition, chondrocytes in these co-cultures showed reduced expression of mRNA encoding collagen II α1 chains, and increased gene expression of MMP3 and ADAMTS-4 and 5. These effects of osteoblasts in co-culture were largely blocked by neutralizing interleukin (IL)-6 antibodies. These results suggest, first, that osteoblasts obtained from the sclerotic subchondral bone from OA patients somehow retain an OA phenotype after removal from the body and expansion, and culture *ex vivo*. Second, although it is not possible to extrapolate from these *in vitro* findings to the *in vivo* situation, nonetheless there is at least the possibility that aberrant expression of molecules in OA by, in this case osteoblasts, can affect chondrocyte behavior. Osteocytes are the most abundant cell type in bone and are the primary mechano-sensing cell type.^29,30^ The osteocyte lacuna–canalicular network is in functional continuity with the bone micro-vasculature.^31^ As discussed above, osteocytes in the subchondral bone are closely juxtaposed to the cartilage, and can potentially signal into the cartilage via the lacuna–canalicular network or the bone micro-vasculature. Very little has been reported concerning this potential interaction; however, Priam *et al.*^32^ conducted intriguing experiments with mouse calvarial osteoblasts/osteocytes. The cells were subjected to cyclic compression, after which the conditioned medium was collected and incubated with mouse articular chondrocytes. Conditioned medium from the ‘loaded’ cells caused a marked upregulation of MMP3 and MMP13 expression in the chondrocytes and downregulation of the expression of aggrecan and type II collagen. The study identified 14-3-3ε as a soluble mediator for communication between the osteoblasts/osteocytes and chondrocytes.^32^ This study shows the possibility of mechanical influences in osteoblasts secondarily affecting articular chondrocytes, in addition to direct responses by chondrocytes to load.^20^ Physiological loading is important for cartilage homeostasis and can counter the catabolic effects of inflammatory cytokines.^33^ However, overloading of joints is harmful and persistent overloading is thought to be an important driver of cartilage degradation. It has been speculated that catabolic molecules from diseased cartilage can adversely affect bone cells,^34^ but there are no direct data to show that this occurs *in situ*.

### Crosstalk *in vivo*

There are a number of *in vivo* experiments, which show the inter-dependence of the subchondral bone and articular cartilage compartments in OA induction and progression. These experiments are of two types, namely, those that induce OA by perturbing either the cartilage or bone, but resulting in OA changes in the other compartment, and those where treatments of existing OA, which would be expected to preferentially affect one compartment (in most cases the bone), are protective of OA changes in the other (usually the cartilage). An example of experiments to perturb the cartilage is the injection of vascular endothelial growth factor (VEGF) intra-articularly into the knee joints of mice.^35^ The rationale for these experiments was the consistent finding of increased VEGF in the tissues of OA joints, including cartilage, subchondral bone, and serum.^36^ In this model, exogenous VEGF initiated a full range of osteoarthritic processes in the knee joint, with a temporal sequence of synovial hyperplasia, tidemark duplication in the calcified cartilage and subchondral bone sclerosis, followed by cartilage degradation. The results suggested that perturbations on the articular side of the joint, in this case with VEGF, resulted in changes in all the tissues of the joint, including the subchondral bone. However, it is not clear whether the observations were direct, via signaling from VEGF-treated cartilage to bone, or indirect, via circulating VEGF acting on bone. A second example of intervening from the articular side involves the Wnt inhibitor, Dkk-1.^37^ Members of the Wnt family and their inhibitors are strongly associated with OA, and Wnt signaling pathways have been suggested as potential therapeutic targets.^38^ In this example, the mouse OA model of destablization of the medial meniscus was used to explore the effect of Dkk-1 overexpression in the cartilage, either by adenoviral or transgenic approaches. Although destablization of the medial meniscus caused severe cartilage destruction, osteophyte formation, and subchondral bone sclerosis in control mice, these manifestations of OA were significantly inhibited by Dkk-1 overexpression in chondrocytes. These results show that inhibition of the canonical Wnt pathway by Dkk-1 specifically in articular chondrocytes is sufficient to prevent OA changes in the underlying bone by joint destablization. The mechanism for this is not yet known.

An example of perturbing the subchondral bone, with protective effects for OA development in both bone and cartilage, is a mouse model of osteoblast-specific overexpression of the EphB4 receptor.^39^ The rationale for these experiments was the growing understanding of the role for the ephrin/Eph subfamily of membranous tyrosine kinases in bone biology,^40^ prompting exploration of their role in skeletal pathology. EphB4 transgenic mice were resistant to OA development after destablization of the medial meniscus surgery, compared with control mice. This included the preservation of cartilage and subchondral bone. These findings suggest that maintenance of the metabolism and structure of the subchondral bone following destablization of the joint by destablization of the medial meniscus also protects the integrity of the overlying cartilage. A second example is a mouse model of bone-specific overexpression of TGFβ, which was found to be causal of OA.^41^ The rationale for these experiments was the strengthening case for a role for TGFβ in OA pathogenesis, beginning with the increased amounts of TGFβ protein measured in OA bone^42^ and synovial fluid.^43^ In our own work, elevated levels of TGFβ mRNA were found consistently in bone from individuals with end-stage hip OA,^44^ and cultured human osteoblasts isolated from femoral bone of hip OA patients produced increased TGFβ, which showed altered relationships with the expression of other cell regulatory molecules.^45^ The work by Zhen *et al.*^41^ demonstrated in mice that high concentrations of active TGFβ in the subchondral bone induce abnormal bone formation and the degradation, and loss of the overlying articular cartilage, resulting in OA.^41,46^ These authors produced transgenic mice expressing the CED (Camurati–Engelmann) mutant form of TGFβ1, which is released as active, rather than latent, TGFβ.^47^ Production of the mutant protein specifically by osteoblastic cells led to the activation of Smad2/3 (intracellular downstream TGFβ signaling molecules) in the subchondral bone, but not in the articular cartilage. The mutant mice showed spontaneous OA-like changes in the subchondral bone and degradation of the overlying cartilage.^41^ Evidence for TGFβ acting in the subchondral bone being causal of OA was shown in conventional mouse and rat OA models, in which specific inhibition of TGFβ activity either in the subchondral bone or systemically^48,49^ attenuated the development of OA changes in both the subchondral bone and articular cartilage. These results again show that altered metabolism in the subchondral bone can lead to all the manifestations of OA, including cartilage degradation. As altered TGFβ signaling has subsequently been shown in a spontaneous, age-related model of OA, the Dunkin Hartley guinea pig,^50^ these latter findings may have important implications for human ‘idiopathic’ OA.

Further evidence for the inter-dependence of bone and cartilage comes from the results of experimental treatments for OA. A number of attempts to modify OA progression have focussed on the altered rate of bone remodeling in the subchondral bone. As reviewed by Burr and Gallant,^51^ this changes across the course of the disease, with increased remodeling, accompanied with increased vascularity, characteristic of early OA in animal models of the disease, whereas late-stage disease is characterized by the reduced bone resorption with a bias towards bone formation. Increased bone remodeling, and more specifically increased osteoclast numbers and activity in the subchondral bone,^52^ has been identified as a therapeutic target. The rationale for this is that the resultant changes in bone structure and release of inflammatory cytokines produced by the resorptive process may have biomechanical and biochemical implications for the overlying cartilage. Thus, a number of anti-resorptive agents have been investigated as disease-modifying agents. Examples are alendronate in rabbit models of OA,^53,54^ pamidronate in rat OA,^55^ calcitonin in dog OA,^56^ and osteoprotegerin in mouse OA,^57^ each of which were shown to be chondroprotective. It should be noted that, although the most obvious interpretation for these agents offering cartilage protection is that they do so secondary to inhibiting remodeling events in the underlying bone, there may also be direct actions on chondrocytes. Indeed, there is some evidence for direct cell protective effects of bisphosphonates,^58^ calcitonin,^59^ and osteoprotegerin^60^ on chondrocytes.

Taken together, the evidence cited above shows the close relationship that exists between the articular cartilage and subchondral bone, which in at least some cases may involve a crosstalk. The findings that treatments directed to the subchondral bone are chondroprotective in animal models has been exciting, but attempts to translate these treatments to human OA have been largely disappointing. It is likely that such studies have been confounded by all the factors that make the existing animal models of OA imperfect predictors of human disease. Human OA has a long time course, is episodic, and frequently exists on a background of ageing, obesity, metabolic disturbances, joint malalignment, vitamin D deficiency, and declining sex hormone status.^61^ In addition, although it is easy in animal experiments to start therapy at the time of OA initiation, or at a known time thereafter, this would only be possible in post-traumatic OA in humans. Finally, genetic factors in human populations are likely to hold considerable influence, over which individuals progress to clinically important joint disease, in both traumatic and non-traumatic OA. Genetics determines bone shape, cartilage thickness, and response of bone (and presumably cartilage) to load.^62^ Importantly, as shown for inbred strains of mice, there are strong genetic determinants of articular cartilage repair following tissue injury to the joint.^63^ It is also interesting that in human patients undergoing arthroscopic meniscectomy, a subset of patients showed an articular cartilage gene expression pattern characteristic of OA, despite their macroscopically normal cartilage.^64^ Studies with larger cohorts will be required to determine whether these gene expression patterns are indeed predictive for OA. The study also showed associations between cartilage gene expression patterns and age, body mass index, and sex. Such studies suggest ways to understand the way that risk factors and genetic factors work together to produce the manifestations of OA, and will hopefully enable the targeting of treatments to individuals most likely to respond.

## Changes in cell behavior in OA could alter bone–cartilage crosstalk

Associated with the initiation and progression of OA, a large number of changes occur in the behavior of cartilage and bone cells. This results in the altered expression of many molecules, which could have both autocrine actions in their tissue of production as well as contributing to an altered conversation between the bone and cartilage compartments, given that proteins and other larger molecules are able to transit between these tissues.

There is an extensive literature describing the altered expression profile of chondrocytes in OA. A suite of inflammatory cytokines,^34^ for example, TNFα and members of the interleukin family, influence the function of chondrocytes away from normal homeostasis and anabolism towards a catabolic phenotype. These cytokines arise both externally, for example, from the synovium, or from the traumatized chondrocytes themselves, and stimulate their own expression in a positive-feedback loop, as well as inducing chondrocytes to synthesize MMPs, proteases, chemokines, nitric oxide, and eicosanoids such as prostaglandins and leukotrienes, all of which lead to increased cartilage degradation (reviewed in Houard *et al.*^65^ and Rahmati *et al.*^66^). Zhou *et al.*^67^ recently described the molecular signature of human cartilage derived from different regions of OA knees. They found much higher expression of TNFα, IL-1β, IL-6, IL-11, and IFN-γ in cartilage of OA grade 1–3 (macroscopic outerbridge grade), compared with grade 0, and concomitant increases in ADAMTS-5, MMP-8, and MMP-13, enzymes that degrade the structure of articular cartilage by cleaving the aggrecan and collagen II matrix scaffold. Interestingly, molecules associated with osteoclasts were also elevated in grade 1–3 cartilage, including cathepsin K, TRAP, RANK, and c-fms, as well as stimulators of osteoclast differentiation and function, RANKL and M-CSF. RANKL expression has previously been shown to be elevated in elevated in Osteoarthritis Research Society International (OARSI) grade 2 OA cartilage, predominantly in the pericellular regions of the middle and deep zones of the cartilage.^68^ The authors speculated that RANKL could diffuse from the deep cartilage to contribute to changes in the subchondral bone metabolism.

Evidence for a role for TGFβ in the bony changes of OA is summarized above. TGFβ may also have a role in cartilage in OA progression, although the data so far present a confusing picture, as reviewed by Fang *et al.*^69^ It is reported that TGFβ is present at low levels in mature cartilage, and that its expression and signaling are upregulated in OA. Further, the increased expression of TGFβ links with induction of the matrix-degrading enzyme, HTRA1 in chondrocytes.^70^ It is of interest that a phase II clinical trial in a human OA cohort, in which allogeneic chondrocytes genetically engineered to overexpress TGFβ were injected intra-articularly, reported positive preliminary results for pain and function.^71^ Injury to cartilage also upregulates members of the Wnt family. For example, WNT-16 was found to be highly upregulated in the areas of cartilage damage^72^ and Wnt-3A induced the expression of MMP13 and ADAMTS-4 in primary chondrocyte cultures.^37^ Interestingly, the Wnt antagonists, sclerostin,^73^ and DKK-1^37^ have been shown to protect against cartilage destruction *in vitro* and *in vivo*. Specifically, sclerostin protein was significantly increased in the focal areas of cartilage damage in surgically induced OA in sheep and mice, as well as end-stage human OA, and was shown to be biologically active in chondrocytes. Exogenous sclerostin inhibited the Wnt-β–catenin signaling and catabolic events in cartilage, including IL-1α-stimulated cartilage aggrecanolysis *in vitro*.

A large number of proteins and genes show altered expression in OA bone compared with that in bone from control individuals without OA. In human OA, these studies are limited to sampling at end-stage disease, from which it is difficult to interpolate to disease initiation and progression. Nevertheless, not only have clear differences been found in OA bone compared with that from non-OA individuals, but many of these differences are maintained in cultured osteoblast-lineage cells derived from these bone samples.^74,75^ Kuliwaba *et al.*^76^ measured RNA extracted from the trabecular bone in the intertrochanteric region of the proximal femur, and found reduced expression of IL-6, IL-11, and RANKL in OA bone compared with age-matched autopsy controls. Interestingly, although RANKL mRNA levels associated strongly with the structural parameters Eroded Surface as a percent of Bone Surface (ES/BS) and Osteoid Surface as a percent of Bone Surface (OS/BS) in trabecular bone from control individuals, these relationships were not apparent in OA bone,^77^ suggesting that bone turnover is regulated differently in this disease. Similar findings of reduced expression of RANKL mRNA in OA bone were also reported when comparison was made with bone from femoral neck fracture patients.^78^ Evidence that bone formation is also altered in OA was that osteocalcin mRNA expression was significantly greater in hip OA and, curiously, increased significantly with age in the OA group but not in controls.^76^ Kumarasinghe *et al.*^79^ found not only differences in gene expression between femoral intertrochanteric OA and control bone but also intriguing differences in the relationships between the expression of individual genes and the relationships between gene expression and structural indices (for example, TWIST1 *vs* S100A4: *r*=+0.71, *P*<0.001, *r*=−0.30, *P*=0.20 for OA and control, respectively; CTNNB1 *vs* OS/BS: *r*=−0.07, *P*=0.77, *r*=+0.66, *P*=0.01 for OA and control, respectively). Hopwood *et al.*^44,80^ performed gene microarray analysis on bone from the same region of the femur and identified a large number of differentially expressed genes in OA compared with control or osteoporotic bone. A substantial number of the top-ranking differentially expressed genes are known to have roles in bone formation and, as reviewed by Kumarasinghe *et al.*,^81^ many of these genes are targets of either the Wnt or TGFβ/BMP signaling pathways. These findings are consistent with the roles for these signaling pathways already discussed and with the increased amounts of insulin-like growth factor types I and II, and TGFβ protein measured in OA in bone from the iliac crest.^42^ This latter result, together with the above findings in bone derived from the femoral intertrochanteric region, and other related reports^82^ suggest that bone metabolism may be disturbed throughout the skeleton in OA, rather than these changes being confined to the subchondral bone. If this is the case, several important questions arise: is altered bone metabolism the cause or effect of OA; is it secondary to other factors such as vitamin D deficiency or obesity?

To examine gene expression differences specifically in the subchondral bone in OA, a number of groups have performed gene microarray and PCR analysis in human OA^83^ and animal models of OA.^84,85^ These studies are difficult to interpret but show the differential expression of many genes associated with bone remodeling and mineralization in human end-stage OA subchondral bone. In the animal studies of surgically induced OA, more severe OA manifestations were observed in older animals, accompanied by many more differentially expressed genes in the bone of older animals with OA.

## Enhanced crosstalk associated with BMLs

In human OA, the changes in bone and cartilage vary considerably across the joint, typically being more severe in the medial versus lateral knee joint. Altered remodeling leads to altered bone structure, with increased Bone Volume as a percent of Tissue Volume (BV/TV) in cancellous bone and the formation of osteophytes.^86,87^ Increased trabecular thickness, decreased trabecular spacing, and reduced hardness of the bone in OA,^88^ due to decreased mineral within the bone matrix,^89^ characterize the subchondral bone, particularly in zones of the joint underlying cartilage degeneration. The spatial association of these changes strongly supports the notion of a close functional relationship between subchondral bone and articular cartilage. This relationship is further highlighted by examining bone marrow lesions (BMLs), discrete abnormalities that can be visualized in the subchondral bone using magnetic resonance imaging (MRI; reviewed in Bassiouni,^90^ and Daheshia and Yao^91^). Although commonly observed in symptomatic OA,^92–95^ BMLs are also seen in early (pre-radiographic or asymptomatic) OA.^96–98^ There is growing evidence that BMLs can act as ‘reporters’ of OA, offering prognostic and predictive value for disease progression, and outcome measures for intervention strategies. Most investigations of BMLs have been performed in knee OA, where longitudinal studies have shown that their continued presence is a potent risk factor for structural deterioration, in particular the loss of cartilage volume and quality.^95,99–102^ However, this was also a feature of BMLs in the human femoral head.^103^ Thus, the presence of a BML predicts future joint replacement.^104^

**Figure 1:**
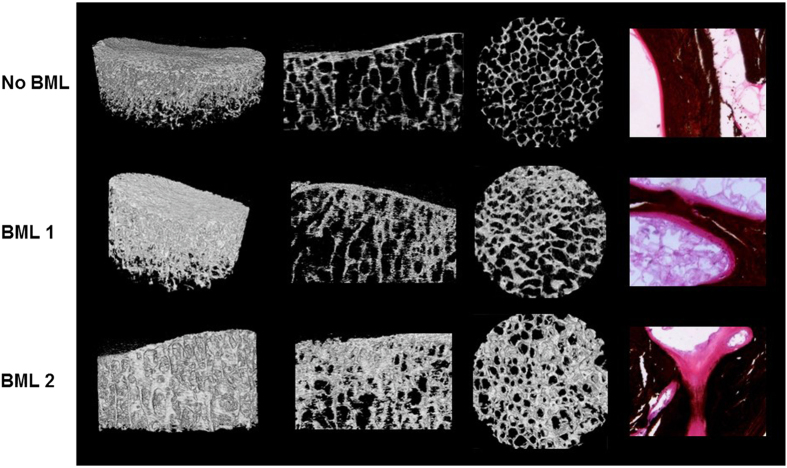
Subchondral bone microarchitecture and osteoid seams characteristic of MRI identified bone marrow lesions (BMLs) in human tibial plateaus obtained at knee arthroplasty surgery for OA. No signal was detected on PDFS and T1-weighted MRI sequences for no-BML. BML signal was detected only by PDFS for BML 1; BML signal was detected by both PDFS and T1 for BML 2. Left panel shows three-dimensional reconstructed micro-computed tomography (CT) images of a 10 mm diameter cylindrical region of interest within the volume of the BML. Left and right middle panels show coronal and axial micro-CT images, respectively. Micro-CT images clearly demonstrate that BML subchondral bone is sclerotic, characterized by thicker subchondral bone plate, and increased trabecular bone volume with more plate-like structure. Right panel shows von Kossa silver/hematoxylin- and eosin-stained subchondral trabecular bone that shows BML tissue has increased osteoid, with increased osteoid seam thickness, and osteoid bridging and in-filling. The sclerotic subchondral bone phenotype characteristic of BMLs was more pronounced for BML 2 versus BML 1.^16^

We have reported on a comprehensive characterization of BMLs at the tissue level, where tibial plateaus were obtained at knee arthroplasty for OA.^16^ BMLs were identified in the subchondral bone *ex vivo* by performing MRI at two clinically relevant sequences—T1- and PDFS-weighted (T2) sequences. BMLs were detected in >70% of tibial plateaus, the remainder comprising a no-BML group and two BML groups were recognized. In the first group, designated BML 1, BMLs were detected only by PDFS (59% of BMLs); in the second group, designated BML 2, BMLs were detected by both PDFS+T1 (41% of BMLs). The tissue within an osteochondral unit defined by a BML was strikingly different from the surrounding tibial tissue. Most importantly, BMLs were associated strongly with degeneration of the overlying cartilage. When compared with no-BML, TP with BML showed bone changes (Figure 1), which included thicker subchondral plate (*P*=0.002), increased trabecular bone volume and plate-like structures (*P*=0.000 4), increased osteoid volume (*P*=0.002) and thickness (*P*=0.005) and osteoid bridging and in-filling (Figure 1), and a striking increase in microcrack density in the subchondral trabeculae (*P*=0.000 1). OA bone showed increased osteoclastic resorption (ES/BS) when compared with the samples of control bone without OA (*P*=0.005), although this parameter changed little across no-BML-BML1, 2, within OA bone. We also found a higher percentage of empty osteocyte lacunae in OA subchondral trabecular bone compared with non-OA controls (*P*=0.05), suggesting that the accumulation of microdamage may lead to a compromised osteocyte network and subsequent osteocyte death, as reported in other circumstances.^23^ BMLs also contained bone marrow changes, which included more bone marrow edema (*P*=0.03), fibrosis (*P*=0.002), necrosis (*P*=0.01), small fibrovascular cyst-like formations (*P*=0.04) (these were not seen in control or no-BML bone), and the increased density of thick-walled arterioles (*P*=0.002). Many of the changes found in BMLs were reminiscent of those for the overexpression of TGFβ in subchondral bone, for example, increased osteoid and vasculature,^41^ suggesting the possible involvement of TGFβ in human OA. In addition, increased recruitment of mesenchymal stem cells was recently described in regions defined as BMLs of the human femoral head,^103^ qualitatively similar to surgically induced and TGFβ-induced OA in mice.^41^ Finally, BMLs in our study were associated with changes in the overlying cartilage, which included reduced cartilage volume (*P*=0.008), tidemark duplication (*P*=0.01), increased vascular cones penetrating into the cartilage (*P*=0.01), and higher OARSI scores (*P*=0.004). For many measures, BML 1 was intermediate between no-BML and BML 2, prompting the provisional conclusion that BMLs detected by specific MRI sequences identify different degrees of degeneration in the osteochondral unit.^16^ This conclusion is supported by a separate study in 297 adults without incident knee pain, in which BMLs present on both T1- and T2 (PDFS)-weighted MRI sequences were associated with significantly increased medial tibial cartilage loss and incident knee pain after 2 years, compared with those BMLs seen only on T2-weighted sequences.^105^ Taken together, this work suggests, first, that MRI imaging of BMLs may enable the identification of different OA phenotypes and more targeted treatments in OA. Second, there was a clear relationship between the presence of a BML in the subchondral bone and cartilage abnormalities. However, because all sampling was at end-stage disease, it was not possible to determine whether changes in one tissue compartment caused changes in the other or whether both bone and cartilage were responding independently to the same tissue insult. It is instructive in this context to note the results of a study, in which a single session of cyclic compressive loading was applied across the knee joint of mice. This led to rapid morphological and cellular alterations in both the subchondral bone and articular cartilage, the latter being persistent.^106^

## Summary and conclusions

**Figure 2:**
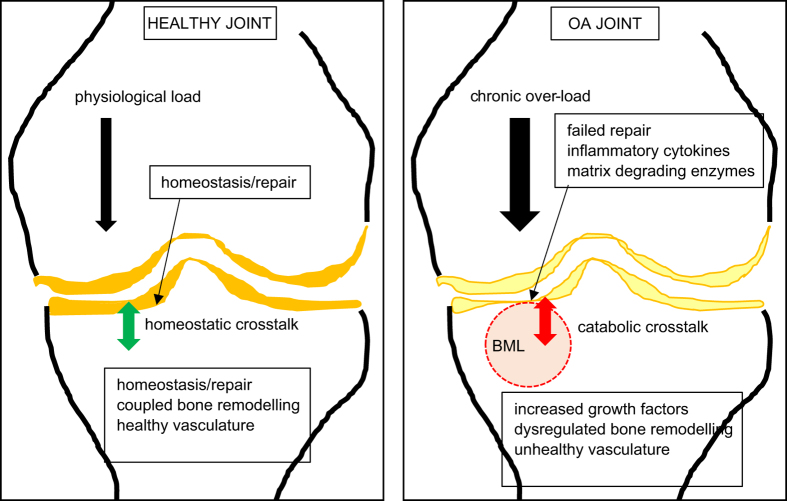
Cartoon of healthy and OA knee joints. Shown is a healthy joint, in which physiological loading (magnitude of load×frequency of loading) is managed by the joint. Healthy subchondral blood vessels and blood perfusion, and a putative healthy crosstalk between the subchondral bone and articular cartilage lead to coupled bone remodeling to maintain homeostasis and repair microdamage in the bone matrix. In the OA joint, chronic overloading results in failed repair mechanisms. Bone within a bone marrow lesion (BML) zone, imaged by MRI, contains the most severe bony manifestations, in which dysregulated bone remodeling results in both bone attrition and sclerosis. The cartilage overlying the BML is degraded, with increased matrix proteases and decreased production of cartilage matrix. A putative unhealthy crosstalk between the bone and cartilage compartments exacerbates the failure of repair mechanisms, in the face of continued unfavourable biomechanics.

The major points of the review are summarized in Figure 2, including the putative role of crosstalk between subchondral bone and cartilage, as both a means of maintaining the health of the joint and a disease mechanism in OA. The studies summarized above show the feasibility of such crosstalk. However, with respect to a role for this crosstalk in the development of human OA, many questions remain. Do signaling molecules from bone and cartilage traverse between these tissue compartments? If so, is this a cause or a result of OA? Are the reported animal and *in vitro* studies informative of human OA? How can we obtain better data for human OA earlier in the disease process? Are molecules such as VEGF and TGFβ, which appear to be key drivers of mouse OA by acting in one or both compartments, also central players in human OA? If so, how can this knowledge be exploited to treat human OA and how could responsive subjects be identified? There is a clear need for longitudinal studies in human OA, using high-resolution modalities such as MRI, to better understand this disease and that will enable stratification of human subjects into those who are likely to progress, or heal, or be responsive to treatment. The above data allow cautious optimism that we are approaching this point in the history of OA research.
